# COVID-19 Infection Presenting as an Isolated Severe Acute Liver Failure

**DOI:** 10.7759/cureus.24873

**Published:** 2022-05-10

**Authors:** Junaid Khawaja, Aditi Bawa, Hanan Omer, Farooq Ashraf, Palwasha Zulfiqar

**Affiliations:** 1 Internal Medicine, University of Pittsburgh Medical Center (UPMC) McKeesport, Pittsburgh, USA; 2 Internal Medicine, Gujranwala Medical College, Gujranwala, PAK

**Keywords:** omicron variant, covid induced ards, decompensated liver failure, acute liver failure (alf), covid-19

## Abstract

The clinical features of severe acute respiratory syndrome-coronavirus disease 2019 (SARS-COVID-19) infection range from mild upper respiratory symptoms to severe acute respiratory failure. Among other less common features are diarrhea, nausea, vomiting, elevated liver enzymes, and acute kidney injury. We present a case of a 49-year-old female with no preexisting liver disease who presented with weakness and dizziness for one week. Initial investigations revealed acute liver failure (ALF) and positive COVID-19 on polymerase chain reaction (PCR) testing. The patient did not have any upper or lower respiratory symptoms, and extensive workup to look for other etiologies of acute liver failure was unremarkable. She eventually deteriorated to decompensated liver failure and was transitioned to comfort measures only.

Liver injury is a well-documented phenomenon associated with COVID-19 infection. Some of the common pathophysiological mechanisms include direct liver injury, immune-mediated liver damage due to the severe inflammatory response, ischemic injury, endothelial disruption, and coagulopathy. Our case uniquely highlights that SARS-COVID-19 infection may have the potential to solely affect hepatocytes without the classic severe acute respiratory distress syndrome. This case demonstrates that a diagnosis of COVID-19 may be considered if no other etiology of ALF is identified.

## Introduction

As of April 2022, five hundred million cases of coronavirus disease 2019 (COVID-19) infection have been reported worldwide [1]. COVID-19 infection continues to evolve into various strains, with the most predominant being the Omicron variant at the time of this case report [2]. The clinical features and severity of COVID-19 infection differ based on age and other co-morbidities such as diabetes, obesity, hypertension, and underlying liver disease [3]. The most common symptoms associated with COVID-19 infection include sore throat, fever, cough, anosmia, headaches, diarrhea, nausea, and vomiting.

A literature review reveals extensive evidence of COVID-19-induced acute liver injury (ALI). For example, some studies have demonstrated a strong correlation between COVID-19 severity and liver injury [4] with outcomes depending upon the degree of liver injury. In a large cohort study done on 2,273 patients in the United States, COVID-19 was associated with a mild degree of ALI, however, 6.4% developed severe ALI and had poor outcomes [4]. It is important to mention that patients who had severe ALI also had higher rates of severe acute respiratory distress (65%) and renal failure (33%) [4].

Our case uniquely highlights the occurrence of COVID-19 infection, which can present with isolated severe ALI without any other systemic organ involvement. Though our patient did have acute kidney injury (AKI) on presentation, it was attributed to hepatorenal syndrome. In our patient, a direct causality of COVID-19 with ALI cannot be established since a liver biopsy was not performed, but extensive workup for other ALI etiologies was negative, making COVID-19-induced severe liver injury a diagnosis of exclusion. More research and investigations are needed to establish a direct correlation between isolated severe acute liver injury and COVID-19 infection, especially in the presence of evolving COVID-19 strains [1].

## Case presentation

**Figure 1:**
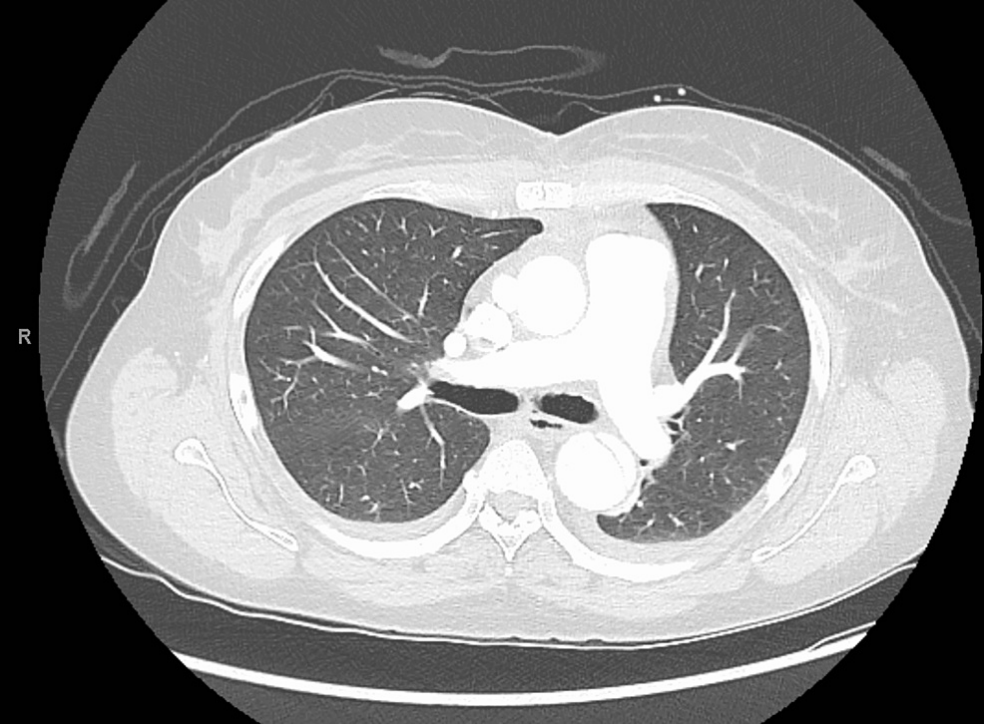
CT chest done in the emergency department showing no acute cardiopulmonary pathology

A 49-year-old female with a past medical history notable for type A aortic dissection status post repair complicated by type B residual dissection, hypertension, and prior benzodiazepine/cocaine abuse presented to the emergency department complaining of weakness for a week and experiencing a fall at home secondary to dizziness. Vital signs on presentation were recorded as heart rate 85, respiratory rate 22, blood pressure 145/90, and temperature 37.8-degree celsius. An initial pertinent physical exam showed normal bowel sounds and no abdominal tenderness or distension. Given the patient's history of type A aortic dissection, a CT angiogram of the chest (Figure 1) and abdomen pelvis was performed, which revealed stable type B dissection extending from the aortic arch to the left common iliac artery.

Laboratory examination results, over the course of the hospital stay, are shown in Table 1.

**Table 1:** Laboratory results over the course of the hospital stay WBC, white blood cell; RBC, red blood cell; eGFR, estimated glomerular filtration rate; INR, international normalized ratio

Laboratory results	Day 1	Day 2	Day 3	Day 4	Day 5	Day 6	Day 7	Day 8
WBC count (4.8-10.8 K/uL)	11.1	10.5	9.6	8.6	10.3	10.4	9.8	4.9
RBC count (4.7-6.1 M/uL)	5.36	4.87	4.36	3.22	2.94	2.91	2.86	2.97
Hemoglobin (14-18 g/dL)	15.7	14.2	13.7	12.7	9.7	8.2	8.6	8.9
Platelet count (13-400 K/uL)	149	106	96	91	52	30	26	20
Serum albumin (3.5-5.2 mg/dL)	2.5	2.0	2.4	2.2	2.7	3.3	3.2	2.8
Total bilirubin (0.2-1.2 mg/dL)	12.96	12.82	15.6	15.2	18.3	21.2	12.3	13.4
Alkaline phosphatase (30-115 U/L)	187	174	151	122	100	59	68	94
Aspartate aminotransferase (0-41 U/L)	950	878	654	706	486	293	113	124
Alanine aminotransferase (0-41 U/L)	1375	1183	823	677	524	433	104	74
eGFR (≥60 mL/min/1.73 m2)	46	42	36	30	42	46	30	32
INR	4.2	5.5	6.0	15.5	11.8	13.1	7.3	12.6
Fibrinogen (mg/dL) (204-570)	60				156			
Ammonia (9-33 uMol/L)	52	102	139	68	112	132	66	102
Lactate (0.5-2.2 uMol/L)	3.4	4.5	3.2	6.3	5.6	6.1	5.9	4.0

She denied a history of cirrhosis or other liver problems. She also denied any recent use of illicit drugs, herbal products, excessive alcohol, or large ingestions of acetaminophen or aspirin. Ethanol level, toxic alcohol, urinary drug screen, salicylate level, acetaminophen level, hepatitis panel, including hepatitis E, thyroid-stimulating hormone (TSH) level, ceruloplasmin level, cytomegalovirus (CMV)/Epstein-Barr virus (EBV) titers, anti-nuclear antibody (ANA), anti-mitochondrial antibody (AMA), anti-smooth muscle antibody (ASMA), liver-kidney microsomal antibodies (LKM) - all were negative. Abdominal ultrasound with Doppler showed patent circulation and was negative for thrombosis, cirrhosis, or biliary disease. As her liver function tests and mental status continued to deteriorate, a decision was made to transfer the patient to a tertiary center equipped with a transplant facility. Prior to the transfer, a protocol-based COVID-19 polymerase chain reaction (PCR) test returned positive.

At the tertiary care center, the patient was treated with lactulose and rifamixin for hepatic encephalopathy (HE), intravenous (IV) vitamin K for coagulopathy, IV 25% albumin for the hepatorenal syndrome (HRS), and empiric N-acetylcysteine for acetaminophen toxicity or ALF. Over the course of the next four days, she had worsening hepatic encephalopathy (HE), hepatorenal syndrome (HRS), thrombocytopenia, a rapid increase in prothrombin time/INR unresponsive to fresh frozen plasma, vitamin K, and platelet transfusions. High-volume plasma exchange therapy was also initiated to manage ALF. On the fifth day of the hospital course, she developed acute hypoxemic respiratory failure requiring emergent intubation. The possible etiologies were volume overload secondary to renal failure or acute respiratory distress syndrome (ARDS) secondary to aspiration. Owing to multiorgan failure with no clinical improvement and poor prognosis, the patient was transitioned to comfort measures on the ninth day of the hospital course and she peacefully passed away. 

## Discussion

The SARS-COVID-19 pandemic has become an epicenter of public health crisis since the end of 2019. It has evolved into various strains, the notable ones being delta, omicron, and BA.2 variants. A usual clinical course of COVID-19 infection involves upper respiratory infection, fever, chills, and anosmia, followed by viral pneumonia, severe acute respiratory distress syndrome, and multiorgan failure [3]. Recently, there have been few case reports of COVID-19 infection in children presenting as severe acute hepatitis [5]. Our report describes a case of an adult woman presenting with severe ALF as her only presenting sign and symptom of COVID-19 infection.

Literature review reveals extensive evidence of the COVID-19 virus affecting liver cells, ranging from mild ALF to moderate and severe ALF. The proposed pathophysiology involves direct COVID-19-induced cytotoxicity, coagulopathy, severe inflammatory reaction, ischemic liver injury due to severe respiratory distress, drug-induced liver injury due to drugs like remdesivir, or liver congestion secondary to right-sided heart failure [6]. COVID-19 virus has been isolated from liver biopsies in 15 out of 22 samples through in situ hybridization as demonstrated in a study done by Aurelio Sonzogni et al. [7]. Further, this study demonstrated extensive portal vein thrombosis, liver fibrosis, and hepatic vein dilations on postmortem samples of liver biopsies in 48 COVID-19 patients [7]. In vitro studies have demonstrated the interaction of angiotensin-converting enzyme 2 (ACE2) receptor on hepatocytes with spike protein of COVID-19 virus as the primary mechanism of direct cytotoxic injury [6]. However, the most common pathophysiology of liver injury in COVID-19-infected patients seems to be ischemia secondary to severe ARDS [6].

We considered the possibility of other pathologies that may explain her acute liver failure. Our patient had a history of aortic dissection and type-B residual dissection, however, a chest CT angiogram on presentation showed stable Type-B residual dissection. Furthermore, an abdominal ultrasound with Doppler showed patent liver circulation. Similarly, we considered the possibility of drug overdose or intoxication; however, the patient’s urinary drug screen, comprehensive drug screen, ethanol, and acetaminophen levels were negative. She also denied any drug overuse or recent binge drinking. Lastly, we also considered the possibility of drug-induced liver injury; especially with the advent of remdesivir and other COVID-19 anti-virals, however, our patient did not receive any of these drugs.

Our case highlights the presence of severe ALF in the absence of viral pneumonia or ARDS. A liver biopsy could not be performed, as the patient was unstable and hypo-coagulable, however, an exhaustive workup for other causes of ALF was negative. Considering recent case reports showing COVID-19 infection as a direct cause of acute hepatitis in children [5], our case report emphasizes the importance of further research into the possible association between COVID-19 and isolated ALF.

## Conclusions

In the current COVID-19 pandemic with evolving variants, a diagnosis of COVID-19 should be considered in a patient presenting with acute liver failure of unknown etiology. There have been numerous case reports of children presenting with an isolated ALF who were diagnosed with COVID-19 infection. To our knowledge, this is the first case report of an adult woman with an isolated severe ALF in the presence of COVID-19 infection. Though a causality between COVID-19 and ALF cannot be established, further research is necessary to explore the direct effects of COVID-19 on liver cells.
